# Heats of Hydrolysis and Formation of Potassium Borohydride

**DOI:** 10.6028/jres.065A.011

**Published:** 1961-04-01

**Authors:** Walter H. Johnson, Richard H. Schumm, Isa H. Wilson, Edward J. Prosen

## Abstract

The heat of reaction of potassium borohydride with 0.060 molal HCl has been measured by solution calorimetry. The heat of solution of the hydrolysis products has also been measured and combined with certain literature values to calculate the process:

$$\begin{array}{l} {\text{KBH}}_{4}(c)+\text{HCl}\;(g)+3H_{2}\text{O}\;(\text{liq})\to \text{KCl}\;(c)+H_{3}{\text{BO}}_{3}(c)+4H_{2}(g),\\ \Delta H{}^{\circ}\left(25{}^{\circ}C\right)=-354.06\pm 1.84\;\text{kj}/\text{mole}\;(-84.62\pm 0.44\;\text{kcal}/\text{mole}). \end{array}$$

A combination of this value with literature values for the heats of formation of HCl(g), H_2_O(liq), KCl(c), and H_3_BO_3_(c) gives for KBH_4_(c):

ΔHf°(25°C)=−228.86±2.30kj/mole(−54.70±0.55kcal/mole).

Other data on the heats of formation of the alkali-metal borohydrides are discussed briefly.

## 1. Introduction

The metallic borohydrides constitute a unique class of chemical compounds that contain the borohydride group, BH_4_^−^. A number of borohydrides have been prepared; some are ionic in character and others are covalent. The alkali-metal borohydrides have ionic structures and are much more stable toward oxygen and moisture than are the covalent compounds.

The reactivity of the alkali-metal borohydrides toward moisture decreases with increase in the atomic weight of the metal. Potassium borohydride does not form a hydrate as does the sodium salt, and, although quite soluble, it hydrolyses very slowly in water. In dilute acid, however, the hydrolysis is rapid and quantitative, with the liberation of 4 moles of hydrogen per mole of the hydride.

Combustion-calorimetric methods are generally not applicable to the borohydrides because of uncertainties in the composition and thermodynamic states of the combustion products. Nathan, Stegeman, and Mason [1]^1^ investigated the combustion of LiBH_4_ and found that the products included LiBO_2_ together with significant amounts of elemental boron, Li_2_O, and B_2_O_3_. For this reason, we have used solution reactions to obtain the heat of formation of potassium borohydride.

## 2. Materials

The KBH_4_ was obtained from Metal Hydrides, Inc. Analysis by R. A. Paulson of the Analytical Chemistry Section of the Chemistry Division gave 98.79, 99.28, and 99.33 weight percentages (±0.09) of theoretical hydrolyzable hydrogen, boron, and potassium, respectively. Thus, the molar ratio of KBH_4_ to total B was 0.9951. The impurities were probably KBO_2_ and H_2_O or their equivalent in partially hydrolyzed sample. Douglas and Harman [2], who measured the heat content of samples taken from the same lot of material, have given details of the analytical procedures and the results of exposure to moist air.

The KCl and H_3_BO_3_ were reagent-grade materials, dried in vacuo over anhydrous magnesium Perchlorate. The hydrochloric acid solution was prepared by dilution of reagent-grade acid; the concentration was determined by titration with a standard alkali solution.

## 3. Apparatus and Procedure

The calorimeter was similar to that previously described [3], except that the liquid capacity was 400 ml and the resistance of the calorimeter heater was 228 ohms. The cooling constant was 0.0010 °C min^−1^ (°C of thermal head).^−1^ The rise in temperature attributed to stirring energy and bearing friction was 0.0003 °C/min.

The thermometric system and the apparatus for measurement of electrical energy were similar to those previously described [4, 5], except that a White double potentiometer was used for the potential measurements.

Samples of approximately 0.01 mole of KBH_4_ were placed in thin-walled, soft-glass bulbs and dried for 16 hours at room temperature over anhydrous magnesium Perchlorate in an evacuated desiccator. The sample bulbs were then removed, connected to a suction tube, and sealed under a partial vacuum.

The KCl-H_3_BO_3_ mixtures were prepared by weighing 0.010 mole of each of the air-dry materials into glass bulbs, which were then dried and sealed as described above.

The calorimetric solution, 398 g of 0.0597 molal HCl, was adjusted to 23.5 °C and weighed in the calorimeter. The stirrer, the calorimeter heater, the KBH_4_ sample, and the bulb-crushing apparatus were lowered into the calorimeter, and the platinum resistance thermometer was inserted. The assembled calorimeter was placed in a water bath which was thermostatically controlled at 25.2 °C within ±0.04 °C. Calorimeter temperatures were observed at 2-min intervals during a 20-min initial rating period, after which the sample bulb was broken. Observations of calorimeter temperature were continued at 1-min intervals during a 15-min “reaction” period and at 2-min intervals during a 20-min final rating period.

The hydrogen, which evolved quite rapidly, passed through a vent in the calorimetric vessel and was collected in a collapsed rubber balloon that had previously been flushed with dry helium. The balloon distended without causing any appreciable increase in pressure. The vapor mixture in the balloon was next drawn slowly through a weighed water-absorption tube containing anhydrous magnesium Perchlorate and phosphorus pentoxide. The balloon was then refilled with dry helium and emptied through the water-absorption tube. The increase in weight of the tube gave a fairly accurate measure of the quantity of water which vaporized from the calorimetric solution.

The quantity of boric acid in the resulting solution was determined by neutralization followed by titration with standard alkali solution in the presence of d-mannitol; a Beckman *p*H-meter was used to determine the end-point of the titration. The amount of reaction is based on this titration.

The calorimetric system was calibrated with electrical energy, over approximately the same temperature interval, as previously described [4, 5].

The calorimetric system used for the heat of solution of the KCl-H_3_BO_3_ mixture differed slightly from that used for the KBH_4_ hydrolysis experiment in that the concentration of HCl was changed to 0.0298 molal, the calorimeter vent was closed, and the initial calorimeter temperature was 25.0 °C.

## 4. Results and Calculations

All atomic weights are taken from the 1957 International Table of Atomic Weights [6]. The unit of energy employed is the joule; for conversion to the conventional thermochemical calorie, one calorie is taken as 4.1840 joules. All instruments were calibrated with reference to standards maintained at NBS.

The results of the electrical calibration experiments are given in table 1. The energy equivalent of the system, *E_s_*, is obtained as the ratio of the electrical energy, *E*, to the corrected temperature rise, Δ*Rc* [7]. A correction, Δ*e*, is applied to correct the energy equivalent of the actual calorimetric system to a selected “standard” system. The deviations from the standard calorimetric system were due to small variations in the mass of solution.

The results of the experiments on the hydrolysis of KBH_4_, table 2, are calculated by

$$-\Delta H\left(25{}^{\circ}C\right)=\frac{\Delta Rc(E_{s}+\Delta e)+q_{v}}{{\text{mole}\;\text{H}}_{3}{\text{BO}}_{3}\times 0.9951}-0.29.$$

The quantity 0.9951 is the molar ratio of B hydrolyzable as KBH_4_ to total H_3_BO_3_ produced as calculated from the analytical data. The quantity 0.29 kj/mole is the estimated heat liberated,

$$\frac{\left(0.9928-0.9879)\;(14.1)\;(4.1840\right)}{0.9879\;}\text{kj}/\text{mole},$$

by the KBO_2_ impurity accompanying one mole of KBH_4_. The Δ*H* of reaction of KBO_2_ under the conditions of the experiment, −14.1 kcal/mole, is obtained from its heat of formation, −233 kcal/mole [9], and other appropriate data [8]. The correction, *q_v_*, applied for the vaporization of water from the calorimeter solution, is calculated from the mass of water collected in the absorption tube, using 2440 j/g for the heat of vaporization. The heat capacity of crystalline KBH_4_ is taken as 23.03 cal/mole °C [2].

Thus, for the process:

$$\begin{array}{l} {\text{KBH}}_{4}\left(c\right)+\left[2.20\;\text{HCl}+2053\;H_{2}O\right]\left(\text{soln}\right)\to \\ \left[\text{KCl}+H_{3}{\text{BO}}_{3}+1.20\;\text{HCl}+2050{\;\text{H}}_{2}O\right]\left(\text{soln}\right)+4H_{2}\left(g\right)\\ \Delta H\left(25\;{}^{\circ}C\right)=-240.96\pm 1.76\;\text{kj}/\text{mole}\left(-57.59\pm 0.42\;\text{kcal}/\text{mole}\right). \end{array}$$ (1)

The results of the experiments on the heat of solution of a mixture of .01 mole each of KCl and H_3_BO_3_ in 2206 moles H_2_O containing 1.18 moles HCl are given in table 3. A correction of 27.9 j/ohm in the energy equivalent of the calorimetric system is made for the change in concentration of the hydrochloric acid solution. Ignoring the negligible heat effect accompanying small changes in concentration [8], we have:

$$\begin{array}{l} \text{KCl}\left(c\right)+H_{3}{\text{BO}}_{3}\left(c\right)+\left[1.20\;\text{HCl}+2050\;H_{2}O\right]\left(\text{soln}\right)\to \\ \;\left[\text{KCl}+H_{3}{\text{BO}}_{3}+1.20\;\text{HCl}+2050{\;\text{H}}_{2}O\right]\left(\text{soln}\right),\\ \;\Delta H\left(25\;{}^{\circ}C\right)=38.57\pm 0.64\;\text{kj}/\text{mole}\left(9.22\pm 0.15\;\text{kcal}/\text{mole}\right). \end{array}$$ (2)

The heats of solution of gaseous HCl, in water, are calculated from existing data [8] for the following processes:

$$\begin{array}{l} 2.20\;\text{HCl}\left(g\right)+2053\;H_{2}O\left(\text{liq}\right)\to \\ \;\left[2.20\;\text{HCl}+2053{\;\text{H}}_{2}O\right]\left(\text{soln}\right),\\ \;\Delta H\left(25\;{}^{\circ}C\right)=-39.261\pm 0.040\;\text{kcal}/\text{mole} \end{array}$$ (3)

$$\begin{array}{l} 1.20\;\text{HCl}\left(g\right)+2050\;H_{2}O\left(\text{liq}\right)\to \\ \;\left[1.20\;\text{HCl}+2050{\;\text{H}}_{2}O\right]\left(\text{soln}\right),\\ \;\Delta H\left(25\;{}^{\circ}C\right)=-21.448\pm 0.020\;\text{kcal}/\text{mole}. \end{array}$$ (4)

The appropriate combination of equations 1, 2, 3, and 4 yields the process:

$$\begin{array}{l} {\text{KBH}}_{4}\left(c\right)+\text{HCL}\left(g\right)+3H_{2}O\left(\text{liq}\right)\to \text{KCI}\left(c\right)+H_{3}{\text{BO}}_{3}\left(c\right)+4H_{2}\left(g\right),\\ \Delta H{}^{\circ}\left(25\;{}^{\circ}C\right)=-354.06\pm 1.84\;\text{kj}/\text{mole}\left(-84.62\pm 0.44\;\text{kcal}/\text{mole}\right). \end{array}$$

The heat of formation of crystalline boric acid is taken as −262.16 ± 0.32 kcal/mole [9]. This value, combined with the literature values [8] for the heats of formation of KCl(c), HCl(g), and H_2_O(liq), gives for KBH_4_(c):

ΔHf°(25°C)=−228.86±2.30kj/mole(−54.70±0.55kcal/mole).

The uncertainties assigned to the experimental values given in this paper are determined by combining twice the standard deviation of the mean of the experimental data with reasonable estimates of all other known sources of error.

## 5. Summary

No previous experimental data on the heat of formation of KBH_4_ have been reported. Altshuller [10] estimated the heats of formation of KBH_4_, RbBH_4_, and CsBH_4_ to be −58, −59, and −63 kcal/mole, respectively, from lattice-energy calculations. Davis, Mason, and Stegeman [11] measured the heats of hydrolysis of LiBH_4_ and of NaBH_4_ in aqueous hydrochloric acid, using a closed system, and obtained −44.15 and −43.83 kcal/mole, respectively, for the standard heats of formation. The available data on the heats of formation of the alkali-metal borohydrides are summarized in the following tabulation in kcal/mole at 25 °C:
LiBH_4_−44.15[11]NaBH_4_−43.83[11]KBH_4_−54.70±0.55This investigationRbBH_4_−59[10]CsBH_4_−63[10]

## Figures and Tables

**Table 1:** Results of the electrical calibration experiments

Experiment No.	Δ*Rc*	*E*	Δ*e*	*E_8_*
				
	*Ohm*	*j*	*j/ohm*	*j/ohm*
1	0.155645	2813.14	35.2	18038.9
2	.155159	2810.21	35.2	18076.6
3	.155531	2805.02	−13.7	18048.8
4	.154673	2797.75	19.1	18069.0
	
Mean				18058.3
Standard deviation of the mean				±8.7

**Table 2:** Results of the experiments on the hydrolysis of *KBH_4_*

Experiment No.	Δ*Rc*	Δ*e*	*q_v_*	H_3_BO_3_	−Δ*H*(25 °C)
					
	*Ohm*	*j/ohm*	*j*	*Mole*	*kj/mole*
1	0.137899	−2.5	104.44	0.01074	242.47
2	.162020	8.4	111.62	.01255	243.04
3	.124036	3.5	173.55	.01015	238.67
4	.131172	47.8	189.77	.01074	239.70
5	.120168	41.2	133.89	.009619	240.94
	
Mean					240.96
Standard deviation of the mean					±0.82

**Table 3:** Results of the experiments on the solution of the *KCl — H_3_BO_3_* mixtures

Experiment No.	Δ*Rc*	Δ*e*	KCl–H_3_BO_3_	−ΔH (25°C)
				
	*Ohm*	*j/ohm*	*Mole/each*	*kj/mole*
1	−0.021580	−0.9	0.01000	−39.03
2	−.021260	−6.1	.01000	−38.44
3	−.021146	−5.0	.01000	−38.23
	
Mean				−38.57
Avrrage deviation from the mean				±0.31

## Footnotes

1 Figures in brackets indicate the literature references at the end of this paper.
